# Unweaving the mitotic spindle: A focus on Aurora kinase inhibitors in lung cancer

**DOI:** 10.3389/fonc.2022.1026020

**Published:** 2022-10-27

**Authors:** Alessio Stefani, Geny Piro, Francesco Schietroma, Alessandro Strusi, Emanuele Vita, Simone Fiorani, Diletta Barone, Federico Monaca, Ileana Sparagna, Giustina Valente, Miriam Grazia Ferrara, Ettore D’Argento, Mariantonietta Di Salvatore, Carmine Carbone, Giampaolo Tortora, Emilio Bria

**Affiliations:** ^1^ Comprehensive Cancer Center, Fondazione Policlinico Universitario Agostino Gemelli, IRCCS, Rome, Italy; ^2^ Section of Medical Oncology, Università Cattolica del Sacro Cuore, Rome, Italy

**Keywords:** lung, cancer, oncology, Aurora kinase (AURK), mitosis, AURK inhibitors

## Abstract

Lung cancer is one of the most aggressive malignancies, classified into two major histological subtypes: non-small cell lung cancer (NSCLC), that accounts for about 85% of new diagnosis, and small cell lung cancer (SCLC), the other 15%. In the case of NSCLC, comprehensive genome sequencing has allowed the identification of an increasing number of actionable targets, which have become the cornerstone of treatment in the advanced setting. On the other hand, the concept of oncogene-addiction is lacking in SCLC, and the only innovation of the last 30 years has been the introduction of immune checkpoint inhibitors in extensive stage disease. Dysregulation of cell cycle is a fundamental step in carcinogenesis, and Aurora kinases (AURKs) are a family of serine/threonine kinases that play a crucial role in the correct advance through the steps of the cycle. Hyperexpression of Aurora kinases is a common protumorigenic pathway in many cancer types, including NSCLC and SCLC; in addition, different mechanisms of resistance to anticancer drugs rely on AURK expression. Hence, small molecule inhibitors of AURKs have been developed in recent years and tested in several malignancies, with different results. The aim of this review is to analyze the current evidences of AURK inhibition in lung cancer, starting from preclinical rationale to finish with clinical trials available up to now.

## Introduction

Despite the continuous progress in understanding its biology and discovering new potential targets, lung cancer is responsible for the highest number of cancer-related deaths in Italy (1). Non-small cell lung cancer (NSCLC) represents about 85% of lung cancer new diagnoses and it is a heterogeneous disease, often characterized by the presence of a driver mutation (oncogene-addicted disease) for which a targeted drug is available. The introduction of immune checkpoint inhibitors (ICIs) has changed the history of non-oncogene addicted disease: immunotherapy, alone or in combination with chemotherapy, represents the standard first-line treatment, reaching the biggest benefit in patients with strong expression of PD-L1 (5 years OS: 31.9% vs 16.3% with platinum-based chemotherapy) (2).

Small-cell lung cancer (SCLC) represents the other 15% of lung cancer diagnoses; it is an aggressive disease, with a high proliferation rate and a high dissemination potential, in fact most cases are diagnosed at an advanced stage. Genomic profiling of SCLC identified p53 and pRB as the most frequently altered genes (3), but no targeted therapies are available up to now. Therefore, SCLC is treated as a single entity and platinum-based chemotherapy has been considered the standard of care for the last thirty years. Since the results of IMpower133 and CASPIAN trials, immunotherapy in combination with platinum-etoposide has become the new recommended first-line treatment; although the global benefit of ICIs is small (ΔmOS=2 months), about 15-18% of patients experience a long-term benefit, being alive at 18 months after treatment start (4, 5).

Due to the limited options available after the failure of first-line regimens, particularly in SCLC, research efforts must focus on expanding the therapeutic strategies for lung cancer. An increasing attention has been focused on cell cycle regulators targeting drugs. One of the main actors in cell cycle are Aurora kinases (6, 7). Their importance was initially highlighted by genetic studies on mutants demonstrating their role in the abnormal mitotic spindle formation (from which the name “aurora”, resembling aurora borealis) and cytokinesis failure. In this review, we will focus on the rationale of targeting Aurora kinases in lung cancer, disclosing the results of the available clinical trials.

## Biology of Aurora kinases

Aurora kinases (AURKs) are a family of serine/threonine kinases that plays fundamental roles in cell cycle, particularly in mitotic spindle formation and in chromosome segregation. In mammals, there are three known members of this family: Aurora kinase A (AURKA), Aurora kinase B (AURKB) and Aurora kinase C (AURKC). AURKs are composed of three domains: a N-terminal domain the kinase domain and a C-terminal domain. The catalytic domain shares >70% of homology among the three isoforms (8) and is composed of a β-stranded lobe and an α-helical lobe, linked by a hinge region; the two lobes create a deep cleft where the ATP-binding pocket lies (9). The non-catalytic domains are likewise essential for the correct functions of AURKs: the N-terminal domain mediates the intracellular localization, while the C-terminal domain binds to specific co-factors that shape their conformation (10). The kinase action is only activated after auto-phosphorylation of a specific threonine residue in the catalytic domain.

The specific roles of Aurora kinases depend on the different intracellular localization and the meticulous temporal expression during the cellular cycle. Transcription of AURKs is regulated by cell cycle-dependent factors that bind to cell cycle-dependent elements (CDE) in their promoters (11). AURKC seems to be significantly expressed only in cells undergoing meiosis (i.e., spermatocytes and oocytes) and its biological functions are not well understood. Although it is overexpressed in many cancer types (12), its oncogenic role is unclear; however, it may be responsible for centrosome amplification and multinucleation of cancer cells, conferring survival advantage (13). AURKA and AURKB are, on the contrary, expressed in every cell undergoing mitosis.

AURKA levels rise from G2 phase to early mitotic phases (14–16); at first, AURKA can be found in the pericentriolar matrix and, after activation by co-factor Ajuba, it contributes to centrosome maturation: AURKA recruits several proteins essential to microtubule nucleation, stabilization and spindle assembly, like centrosomin, γ-tubulin ring complex (γ-TuRC) and D-TACC/maskin (17, 18). During late prophase, AURKA phosphorylates cyclin B1-Cyclin-Dependent Kinase 1 (CDK1), which, in turn, provokes the nuclear envelope breakdown (NEBD) by activating the Ran GTPase pathway. After NEBD, AURKA is responsible for centrosome separation by phosphorylating kinesin Eg5, which generates a sliding movement on anti-parallel microtubules pushing the centrosomes away (19). Cyclin B1-CDK1 complex also activates the spindle assembly factor TPX2, which binds to AURKA and, together, they create the bipolar mitotic spindle (20–22).

During early mitosis, AURKB phosphorylates histone H3 in order to release heterochromatin protein 1 (HP-1) from heterochromatin; this event might facilitate chromosome condensation, but evidence is unclear in mammalian cells (23, 24). Then, during prophase, AURKB regulates the attachment of microtubules of mitotic spindle to kinetochores. Kinetochores are protein complexes that bind to chromatin domains which act as a platform called centromeres. AURKB is a member of the error correction (ER) machinery, a control system that detects tension between centromere and kinetochore and stabilizes correct chromosome biorientation (amphitelic), whereas it inhibits incorrect “tensionless” attachments (such as synthelic, monothelic and merotelic) (25). Furthermore, in case of incorrect attachments, AURKB activates the spindle assembly checkpoint (SAC) that prevents sister chromatids separation and mitotic exit (26, 27). During metaphase, AURKB takes part of the chromosome passenger complex (CPC), together with INCENP (inner centromeric protein), Survivin and Borealin, and relocates to the midzone (28). It has been shown in yeasts that AURKB promotes sister chromatid separation by recruiting Shugoshin 1 (SGO1), that removes Cohesin from centromeres (29). Lastly, AURKB plays an essential role in cytokinesis: the activation of RhoA GTPase determines actine polymerization and the formation of the contractile ring; phosphorylation of vimentin, desmin and GFAP creates the cleavage furrow (30).

Given the crucial roles in cell cycle, activity of Aurora kinases must be finely regulated, particularly in case of DNA damages. When G2 checkpoint is activated by double strand breaks, ATM and ATR phosphorylate checkpoint kinase Chk1/Chk2, that not only inhibits cyclin B1, but also AURKA and AURKB; AURKB is also blocked by PARP1 (31, 32).

## Tumorigenic potential of Aurora kinases

Dysregulation of Aurora kinases can lead to proliferative and survival advantages in many tumors. Although there are no validated methods to assess AURK overexpression, different techniques could be used including immunohistochemistry, FISH and comparative multiplex RT-PCR, that can detect differential AURK-mRNA expression in normal and tumor tissues. Overexpression of AURKA is found in different cancers, including lung carcinomas, and is an established poor prognostic factor in lung, breast and colorectal cancers (33–35). The induction of AURKA overexpression *in vitro* did not demonstrate the capacity of transforming cell lines or generating malignant tumors in murine models, so Aurora A might rather be a promoting factor than an oncogene (36). In fact, AURKA overexpressing cells are characterized by multipolar spindle formation and unequal chromosome segregation, leading to aneuploidy and a potentially precancerous state. Moreover, abnormal AURKA activity hyperactivates oncogenic pathways like NFκβ, BCR/ABL and Pi3K/Akt, resulting in increased cell proliferation, survival and transformation. AURKA is also able to upregulate telomerase activity *via* hyperactivation of Myc, leading to increased survival (37). Lastly, AURKA is linked to epithelial-to-mesenchymal transition (EMT) and metastatic potential in several cancer (38, 39). Yoo and colleagues recently showed that AURKA and AURKB confer an “invasiveness signature” in lung adenocarcinoma, indeed their simultaneous inhibition *in vitro* and in a murine model of lung adenocarcinoma reduced tumor invasion (40).

AURKB is found overexpressed in many cancer types (41, 42) and is a negative prognostic factor in NSCLC and hepatocellular carcinoma amongst other tumors (43, 44). Abnormal expression of AURKB is linked to aneuploidy and micronuclei formation, in fact its overexpression alters chromosome segregation and SAC activation (45); in p53-deficient cells, these effects are even augmented (46, 47).

AURKs dysregulation is also responsible for resistance to several antineoplastic drugs. In a recent study by Tagal and colleagues, it was shown that AURKs could determine a switch from the proliferative cell cycle to polyploid growth and multinucleation in lung cancer cell lines, resulting in the formation of polyploid giant cancer cells (PGCC) (48). These cells seem to be associated with resistance to many antimitotic drugs, tumor relapse, immunosuppression, cancer stem cell production, and modulation of the tumor microenvironment (49). Expression of aurora A kinase is correlated with cisplatin resistance in NSCLC: *in vitro* data of 102 NSCLC patients treated with surgery and adjuvant cisplatin-based chemotherapy showed that AURKA expression was elevated in cisplatin-resistant lung cancer cells. Furthermore, its inhibition reversed the migration ability of cisplatin-resistant cells (50). High levels of AURKA are also associated with cisplatin resistance in JAK2-mutated myeloma cells (51).

AURKB’s expression modulates the activity of taxanes in NSCLC cells and the assessment of its levels in histological samples could be developed as a predictive biomarker. It has been shown that mRNA expression of AURKB in NSCLC cell lines inversely correlated with resistance to both docetaxel (p = 0.004) and paclitaxel (p = 0.007). Furthermore, inhibition of AURKB activity with barasertib also demonstrated a strong dose-dependent efficiency in triggering paclitaxel resistance. The results of the study bring to a paradox: overexpression of AURKB reduces survival in chemotherapy-naive patients but, on the other hand, it appears to have a beneficial effect in patients treated with taxane regimens (52).

## Aurora kinases in NSCLC

In a large cohort of NSCLC patients (n = 362) AURKA was highly overexpressed in the tumor tissues compared to corresponding normal lung tissue. In univariate analyses it resulted a significantly increased hazard ratio and poor disease-free survival in patients with a high gene expression of both AURKA (HR = 2.813, p ≤ 0.001) and its co-factor TPX2 (HR = 1.826, p = 0.007). Similarly, AURKA expression confirmed to be a statistically significant prognostic marker using multivariate analyses (p = 0.006) (35).

A study including 11 NSCLC cell lines investigated the preclinical efficacy of MK-5108, a strong inhibitor of AURKA that had shown a potent preclinical activity in malignancies of breast, cervical, colon, ovarian, and pancreatic origin (53). MK-5108 was tested as a single agent and in combination with cisplatin and docetaxel. Concurrent treatment of MK-5108 with cisplatin or docetaxel synergistically inhibited cell growth, with the docetaxel combination performing better. In sequential administration, treatment with docetaxel followed by MK-5108 registered greater growth inhibition than the inverse, even if concurrent treatment remained superior (54).

Different preclinical studies focused on the role of AURKs in oncogene-addicted NSCLC and in particular on their role in the induction of resistance to targeted therapies. Activating mutations in the Epidermal Growth Factor Receptor (EGFR) gene are the most frequent mutations and they can be found in 14–17% of advanced NSCLC in European populations (55). Tumors with common mutations are sensitive to EGFR tyrosine kinase inhibitors (EGFR TKIs), but eventually these patients will develop resistance which will lead to disease progression. Treatment-induced activation of AURKA seems to be associated with *in vitro* and *in vivo* resistance to EGFR inhibitors. In response to chronic EGFR inhibition, AURKA can be activated by the overexpression of TPX2, which facilitate its auto-phosphorylation; TPX2 is normally degraded by a ubiquitin E3 ligase, which is intra-nuclear in both parental and resistant cells (56). In contrast, in resistant cells TPX2 delocalize in the cytosol, separate from the complex responsible for its degradation, leading to its accumulation. Aurora kinase inhibitors suppress this adaptive survival program, increasing the magnitude and duration of EGFR inhibitor response in preclinical models. The suppression of AURKA-driven residual disease could become an important weapon against the acquired resistance in these diseases. The combination of an aurora kinase inhibitor with a third-generation anti-EGFR agent resulted in a synergistic reduction in cell growth in all models (57). In addition, AURKA overexpression is linked to acquired resistance to EGFR-TKI *via* epithelial-mesenchymal transition (EMT), and AURKA inhibitor alisertib has shown to restore NSCLC cells sensitivity to EGFR-TKI and to partially reverse EMT (58). AURKA inhibition with shRNA also demonstrated to partially reverse fibroblast-mediated resistance to gefitinib in EGFR-mutated NSCLC cells co-cultured with stromal cells (59).

Another study showed that resistant EGFR-mutated NSCLC cells without the p.T790M or other acquired mutations are sensitive to AURKB inhibitors barasertib and S49076. In most acquired resistant cells in fact the phospho-histone H3 (pH3), a major product of AURKB, resulted increased and its levels reduced after treatment with AURKB inhibitors, triggering G1/S arrest, polyploidy and, eventually, cell cycle arrest and cell death. The results support the role of AURKB activation in acquired resistance to EGFR TKIs, making AURKB a potential target in NSCLC progressed to anti-EGFR therapy and not carrying resistance mutations (60). AURKB inhibitors are potent enhancers of osimertinib-induced apoptosis and can play an important role in overwhelming acquired resistance to third generation TKIs. Osimertinib resistance caused by EMT activates the ATR-CHK1-Aurora B signaling cascade and generates hypersensitivity to AURKB inhibitors by activating BIM-mediated mitotic catastrophe. AURKB inhibition stabilizes BIM through reduced Ser87 phosphorylation, and transactivates PUMA through FOXO1/3. In this way a combined inhibition of EGFR and AURKB not only efficiently eliminates cancer cells but also overcomes resistance beyond EMT (61).

AURKA and B have also shown to phosphorylate KRAS downstream effectors, playing a synergic oncogenic role with KRAS mutations. Dos Santos et al. demonstrated that KRAS positively modulated AURKA and AURKB expression by regulating their transcription or mRNA stability. They also assessed that simultaneous pharmacological inhibition of AURKA and AURKB activity *in vitro*, as well as their targeting by RNA interference, reduced cell growth and proliferation and promoted apoptosis in a KRAS-dependent manner. Unfortunately, these results were not confirmed in *in vivo* xenografts model; however, this study suggests that aurora kinases could be targeted in KRAS-mutated NSCLC (62). According to results presented at the IASLC 2022 World Conference on Lung Cancer, Lee et al. demonstrated that the addition of AURKA inhibitor VIC-1911 to KRAS inhibitor sotorasib led to increased cell death in resistant cancer cells compared to sensitive ones, suggesting that AURKA inhibition may overcome sotorasib resistance. In addition, the combined inhibition of AURKA and WEE1 led to a synergistic increase in the death of KRAS-mutated lung cancer cells with acquired resistance to sotorasib, even greater than sotorasib plus VIC-1911 (63).

AURK inhibitors were investigated as radiosensitizing agents by Liu et al. in NSCLC cell lines. MLN8237 (alisertib) was assessed together with the effect of radiation and, after treatment, p53-proficient HCC2429 and H460 cell lines increased their sensitivity to the lethal effect of radiation, with a dose enhancement ratio (DER) of 1.33 (*p* < 0.05) and 1.35 (*p* < 0.05), respectively; on the other hand, there was no significant enhanced effect in the naturally p53-deficient and radiation-resistant H1299 cells with a DER of 1.02 (*p* > 0.05). These data suggest that lower doses of radiation could achieve an equivalent antitumor effect when administered in combination with MLN8237 compared to radiation alone *in vitro*, especially in p53-competent cells (64).

Taking into account these early signs of preclinical activity, the role of AURK inhibitors in NSCLC has also been investigated in clinical trials (synthetized in **Table 1**).

**Table 1:** Trials evaluating AURK-I in NSCLC.

FIRST AUTHOR	Type of study	N° of patients (TOT/NSLC)	Drug	Outcome
***Melichar B***	Phase II	249/26	Alisertib	ORR 4% (SD 74%)
***Godwin JL***	Phase I/II	18/18	Alisertib + Erlotinib	ORR 6% (SD 28%)
***Arkenau HT***	Phase I	49/7	AT9283	ORR 0% (SD 29%)
***Semrad TJ***	Phase I	17/5	Alisertib	ORR 0%
***Blakely CM***	Phase I	10/10	Alisertib + Osimertinib	ORR 10% (SD 60%) mPFS 9.4 months
***Mross K***	Phase I	121/4	BI 811283	ORR 0%
***Schoffski P***	Phase II	223/56	Danusertib	ORR 2% PFR at 4 months 10.4% mPFS 9.2 weeks mOS 7.6 months
***Boss DS***	Phase I	59/3	Barasertib	ORR 0%

A multicenter, 5-arm, phase II trial investigated the safety and activity of single-agent alisertib in various advanced and pretreated solid tumors (n = 249). Alisertib was administered orally in 21-day cycles at the recommended dose of 50 mg twice daily for 7 days followed by a break of 14 days. The study included 26 patients with NSCLC and an objective response (OR) was registered in just 1 (4%, 0-22) of 23 evaluable patients, while 17 (74%, 52-90) achieved a stable disease (SD). In the NSCLC cohort, 25 patients (96%) experienced an adverse event (AE) of any grade and the most frequent drug-related grade 3-4 adverse events included neutropenia (62%), leukopenia (27%), fatigue and anemia (both 19%). Despite the manageable toxicity profile, the activity data of alisertib were not particularly promising in patients with NSCLC and did not support further clinical assessment in this disease, in contrast to breast cancer and SCLC (65).

Godwin and colleagues assessed whether the combination of erlotinib and alisertib exerted a synergistic action in EGFR wild-type NSCLC in a phase I/II clinical trial. 18 patients with recurrent or metastatic EGFR wild-type NSCLC were treated and the combination of alisertib and erlotinib proved to be tolerable. Common drug-related adverse events of any grade were fatigue (89%), anemia (83%), leukopenia (78%), dyspnea (78%), diarrhea and anorexia (61%), while drug-related grade 3/4 adverse events included neutropenia and leukopenia (33%), febrile neutropenia, lymphopenia, and anemia (11%). The maximum tolerated dose (MTD) was 150 mg daily for erlotinib with 40 mg BID for alisertib. Disease responses were also noted, including one patient with a partial response who completed 10 cycles, and 5 patients who achieved SD (66).

A single-center phase I study including 17 patients with refractory advanced solid tumors investigated the safety and tolerability of alisertib combined with weekly irinotecan (100 mg/m^2^ on day 1 and 8 of a 21-day cycle). Alisertib was administered orally twice per day on days 1-3 and 8-10 with an escalating dose of 20-60 mg. The MTD was 20 mg twice per day and the dose-limiting toxicities were diarrhea, dehydration, and neutropenia. Furthermore, it was registered one fatal cardiac arrest at the highest dose level tested which was possibly related to drug. No objective responses were observed in patients with NSCLC. Due to the weak activity and most of all to the poor tolerance, the use of alisertib in combination with irinotecan did not show appealing results (67).

Blackely et al. presented at the 2021 ASCO Annual Meeting the promising preliminary results of intermittent dosing of alisertib (30 mg BID on days 1-3, 8-11, and 15-17 of a 28-day cycle) in combination with osimertinib (80 mg daily) in patients with EGFR-mutated lung adenocarcinoma resistant to osimertinib monotherapy. In this phase Ia clinical trial (NCT04085315) 6 patients were treated with 30 mg BID and 4 patients with 40 mg BID intermittent dosing schedule of alisertib. The most commonly reported adverse events were diarrhea (70%), fatigue (60%), alopecia (50%) and neutropenia (50%), all of them of grade 1 or 2; two patients (20%) experienced grade 3 or grade 4 neutropenia, both patients were treated at the 40 mg BID intermittent dose of alisertib. Intermittent alisertib 30 mg BID was identified as the MTD and recommended phase 2 dose in combination with osimertinib 80 mg daily. The ORR was 10% (1/10) and DCR 70% (7/10). The median PFS was 9.4 months (2.0 months - N.R.) (68).

AT9283, an inhibitor of AURKA and AURKB, has been assessed in a phase I dose-escalation study in 49 patients with advanced solid tumors including NSCLC (n = 7). This drug was generally well tolerated with reversible dose-related toxic effects such as myelosuppression, gastrointestinal disturbance, fatigue, and alopecia. No objective responses were observed; however, four patients with esophageal cancer (n = 1), colorectal cancer (n = 1), and NSCLC (n = 2) demonstrated prolonged SD of more than 6 months (69).

The role of another AURKB inhibitor (BI 811283) was investigated in a phase I, dose-escalation study involving 121 patients with advanced solid tumors. The drug was administrated *via* 24-hours infusion on Days 1 and 15 of a 4-week cycle (schedule A) or Day 1 of a 3-week cycle (schedule B) and the MTDs obtained were 125 mg and 230 mg respectively. 4 patients with NSCLC were included in this study: 3 were treated with schedule A and 1 with schedule B. All patients in both treatment schedules experienced at least one adverse event. The most common dose-limiting toxicities were hematological events, particularly neutropenia. Pharmacodynamic assessments showed a decrease in phosphorylated histone H3 (pHH3) which indicated Aurora B kinase inhibition. No patient achieved an OR, even if 30% in schedule A and 33% in schedule B reported a clinical benefit and a stabilization of the disease. Despite a good safety profile, the anti-tumor activity observed does not support the development of the drug in solid tumors (70).

In a prospective, phase II, open-label, multi-institutional study, Danusertib (PHA-739358, a pan-AURK inhibitor) was adopted as single agent for treating patients with different advanced cancers including NSCLC as second line treatment. Patients were treated with danusertib 500 mg/m^2^ given as 24-h i.v. infusion every 14 days until progression or unacceptable toxicity. Danusertib showed marginal antitumor activity with a manageable safety profile. In the 56 patients with metastatic NSCLC the progression-free rate (PFR, the primary outcome) at 4 months was 10.4% (16.1% in squamous subgroup, where the only objective RECIST response was obtained). The mPFS was 9.2 weeks and the mOS 7.6 months. AEs were reported in 83.3% of patients. The most frequent drug-related AEs were fatigue (67.9%), nausea (39.3%), diarrhea (28.6%), anorexia (28.6%), vomiting (16.1%), alopecia (23.2%), constipation (10.7%), anemia and neutropenia (74.5% of events CTC grade 3 or 4) (71).

Barasertib (AZD1152), another Aurora kinases inhibitor, was tested in two phase I studies. Patients with different advanced solid malignancies were treated with escalating doses (100-650 mg) administered as a 2-h infusion every 7 days or 14 days. The MTD was respectively 200 mg and 450 mg, and neutropenia was the most frequent adverse event and dose-limiting toxicity. Grade 3-4 neutropenia occurred in 58% and 43% of patients. No OR were observed at any dose or schedule, although 15 patients (25%) achieved a SD. However, only 3 patients had NSCLC and that is why the role of barasertib is far from being defined in this type of tumor (72, 73).

## Aurora kinases in SCLC

Even after the introduction of immunotherapy in the first-line setting, the majority of patients with SCLC experiences an inexorable disease progression in less than 12 months (4, 5). Unfortunately, effective treatments are not available after disease progression to first-line therapy: topotecan is currently the standard of care, with limited results (74). These poor outcomes highlight the need for a better molecular knowledge of the disease to develop new therapeutic strategies. The most common genetic mutations of SCLC are related to p53 and RB1, but none of these represent a druggable therapeutic target. Amplification of MYC family genes was also found in about 20% of SCLCs (75) and in 30-50% of SCLC cell lines (76) and is associated with treatment resistance, tumor progression and poor outcomes (77, 78). Recent studies have shown that the SCLCs family can be divided into four distinct subtypes based on the differential expression of four transcription factors (79); two of these subgroups, characterized by a high expression of ASCL-1 (SCLC-A) or NEUROD1 (SCLC-N), share a neuroendocrine phenotype; the other two subgroups can be divided on the basis of the expression of POU2F3 (SCLC-P) or of the lack of expression of the three transcription factors (SCLC-I). This last subgroup is instead characterized by the expression of an immunogenic signature, including immune checkpoints and human leukocyte antigens (HLAs), therefore the denomination “inflamed” (80). In a study conducted on murine models, SCLC-N appeared to be associated with MYC amplifications (81, 82). In fact, data suggest that MYC promotes a variant subset of SCLC with lower expression of neuroendocrine markers and with more aggressive features, that could originate from ASCL1+ progenitor cells which, over time, transition to an ASCL1-low/NEUROD1-high state due to the indirect effect of MYC on NEUROD1 signaling (83). Despite these findings, it is still difficult to exploit MYC in a therapeutic way. Nevertheless, from synthetic lethality screenings, AURK inhibitors appeared promising candidate targets. Mollaoglu et al. demonstrated that MYC-driven SCLC cell lines were sensible to AURKA inhibitor Alisertib and AURKB inhibitor Barasertib. To assess AURK inhibition *in vivo*, murine models bearing MYC-amplified SCLC received Alisertib alone, chemotherapy alone or chemotherapy + Alisertib. While single agent alisertib or chemotherapy didn’t show durable results, mice who received the combination had the highest 30-day survival rate (47% vs 5% for chemo-treated vs 8% for Alisertib-treated) (83).

Antitumor activity *in vivo* of these molecules was tested in few clinical trials (synthetized in **Table 2**). A phase I dose-escalation trial tested Danusertib as a 24-hour infusion with and without G-CSF in patients with advanced pretreated solid tumors. Among the 56 patients enrolled in the study, 2 had SCLC. One of these patients experienced an objective tumor response that lasted for 23 weeks receiving 1,000 mg/m^2^ Danusertib + G-CSF, subsequently reduced to 750 mg/m^2^ for hypercreatininemia G2. Drug related SAEs occurred in 21% of all patients (12/56), 9 (22%) in the group treated with Danusertib alone and 3 (19%) in the group treated with Danusertib + G-CSF (84).

**Table 2:** Trials evaluating AURK-I in monotherapy.

FIRST AUTHOR	Type of study	N° of patients (TOT/SCLC)	Drug	Outcome	
***Schoffski P***	Phase II	219/18	Danusertib	ORR 0%, mPFS 8.1 weeks, mOS 11.4 months
***Melichar B***	Phase I-II	249/60	Alisertib	ORR 21%
***Cohen RB***	Phase I	56/2	Danusertib (24h infusion)	ORR 50%
***Carducci M***	Phase I	105/3	AMG 900	ORR 0%

A subsequent multi-cohort phase II study included 18 patients with SCLC who had failed at least two prior lines of therapy that were treated with Danusertib (multi AURK-inhibitor). Unfortunately, none of these patients was progression-free at the four-month treatment assessment. Final results have shown a mPFS of 8.11 weeks and a mOS of 11.4 months. Regarding its safety profile, Danusertib confirmed what had already emerged from previous studies: the most frequent treatment-related non-hematological AEs were asthenia/fatigue (61%, 11/18) and nausea (38.9%, 7/18); neutropenia was the most common hematological toxicity (100%) as well as the most frequent grade 3–4 event (88.9%, 16/18) (71).

A five-arm phase II study investigated the activity of Alisertib in 60 patients with pretreated SCLC. Results have shown that, among response-assessable patients, an OR was obtained in 21% (10/48). The most frequent drug-related grade 3–4 adverse events included neutropenia, leukopenia, and anemia (65).

Lastly, a phase I trial studied AMG 900, an orally administered pan-Aurora Kinase inhibitor in patients with advanced solid tumor. Among the 105 patients treated in this trial, 3 patients of the escalation cohort had SCLC. Unfortunately, none of them obtained an OR with the treatment. Regarding the safety profile, treatment-related AE with grade ≥ 3 occurred in 61 patients (58%); the most common one was neutropenia (n=44, 42%). The most common non hematological AEs were fatigue and diarrhea (85).

The activity and safety of the association of chemotherapy with aurora kinase inhibitors was evaluated in a few clinical trials (synthetized in **Table 3**). In the previously reported phase I study investigating the combination of alisertib and irinotecan in solid tumors, 3 of 17 patients had a diagnosis of SCLC. Although one PR occurred in a patient with SCLC among the 11 evaluable patients (9%), the toxicity profile showed significant rates of toxicities hematological and gastrointestinal toxicities, leading the authors to conclude that the combination of Alisertib and Irinotecan was not well tolerated in adult patients and to stop the planned expansion cohort (67).

**Table 3:** Trials evaluating AURK-I in combination with chemotherapy.

FIRST AUTHOR	Type of study	N° of patients (TOT/SCLC)	Drugs	Outcome
***Semrad TJ***	Phase I	17/3	Irinotecan + Alisertib	ORR 33%
***Graff JN***	Phase 1	41/1	Docetaxel + Alisertib	ORR 0%
***Lim KH***	Phase I	31/5	Nab-paclitaxel + Alisertib	ORR 60%
***Owonikoko TK***	Phase II	178/178	Alisertib/Placebo + Paclitaxel	ORR 22% vs 18%

Another phase I trial in patients with advanced solid tumors tested the combination of Alisertib and nab-paclitaxel, with the rationale of combining their antimitotic action. Among the 31 patients treated in the dose-escalation phase, 5 had a diagnosis of SCLC. Results have shown that one patient with refractory SCLC achieved a partial response that lasted for more than two years, until treatment was discontinued due to neurological toxicities. Two other patients with SCLC achieved a SD that lasted more than four months. These data led to an OR of 6.3% (1/16) and a DCR of 31.3% (5/16) among the 16 evaluable patients. Regarding the safety profile, the most common treatment-related AEs included alopecia (64.5%), diarrhea (41.9%), oral mucositis (41.9%), anorexia (38.7%), fatigue (38.7%), and nausea (35.5%). The most common laboratory abnormalities were leukopenia (80.6%), neutropenia (77.4%) and anemia (77.4%) (86).

A randomized double-blind phase II study assessed paclitaxel + alisertib/placebo as a second line treatment after platinum-based chemotherapy in 178 patients with SCLC, stratified by relapse type (sensitive vs refractory/resistant); mPFS was 3.32 months in the Alisertib + Paclitaxel arm versus 2.17 months in the Placebo + Paclitaxel arm (p=0.113), while mOS was 6.86 months versus 5.58 months (p=0.714). The DCR was 58% in the experimental arm versus 46% in the control arm, and ORR was 22% and 18% respectively. Slightly better results were shown in the subgroup of resistant/refractory patients. In addition, C-Myc-positive patients and those with mutations in genes involved in cell cycle regulation (CDK6, RBL1, RBL2, RB1) also showed better outcomes with Alisertib than with Placebo. The incidence of grade 3 or higher drug-related AEs was 67% with Alisertib + Paclitaxel versus 25% with Placebo + Paclitaxel; the most common AEs were neutropenia, febrile neutropenia, leukopenia, anemia, diarrhea and stomatitis (87).

The combination of Alisertib + Docetaxel was evaluated in a phase I clinical trial in the context of solid tumors eligible for Docetaxel therapy as determined by the investigator. Among the 41 patients that participated, only one patient had a diagnosis of SCLC and did not achieve an objective response. Treatment-related grade 3 or higher AEs involved 39 patients (95%), and the most common one was neutropenia (n=34, 83%) (88).

Lastly, it is worth reporting the case of a nonsmoker patient with SCLC harboring a novel JAZF1-MYCL1 gene fusion and lacking alterations in TP53 and RB1. The patient had previously been treated with chemo-radiotherapy in the setting of limited stage disease; subsequently, after disease recurrence, the patient was enrolled in a clinical trial with Alisertib as his fourth-line regimen and achieved an almost complete response after ten cycles; the patient discontinued treatment after approximately 18 months of therapy (23 cycles) due to disease progression, and after the failure of subsequent chemotherapy lines, obtained an excellent disease control with Nivolumab (89).

## Conclusions

The role of Aurora kinases in regulating cell cycle and safeguarding the correct transmission of genome to daughter cells is well established. Dysregulation of AURKs showed to promote tumorigenesis with different mechanisms, particularly causing aneuploidy and favoring genome instability. In addition, overexpression of AURKs is related to antineoplastic drug resistance, particularly platinum compounds and EGFR-TKIs in the case of lung cancer. Despite the strong rationale in the use of AURK inhibitors against cancer, significant clinical activity was demonstrated in hematological malignancies (90–93) but not in many solid tumors; this different outcome might be explained by the higher proliferation rate and clonality of the formers. In NSCLC, AURK inhibitors showed weak antitumor activity; nevertheless, preclinical studies and early data from clinical studies support their investigation in combination with EGFR-inhibitors. An ongoing clinical trial is evaluating safety and activity of the combination of osimertinib + alisertib or sapanisertib (an oral inhibitor of TOR complex 1 and 2) in osimertinib-resistant EGFR-mutated lung cancer (NCT04479306). Similarly, another clinical trial will study AURKA inhibitor LY3295668 in combination with osimertinib in patients with advanced EGFR-mutant NSCLC who have received a third generation EGFR-TKI (NCT05017025). In SCLC, early-phase clinical trials showed appreciable signals of activity of AURK inhibitors, particularly in combination with taxanes, but these results need to be validated in phase III randomized trials. In addition, considering the better outcomes obtained in cMyc-positive tumors, efforts should be made to apply the concept of precision medicine even in SCLC; the four subgroups based on differential expression of transcription factors ASCL1, NEUROD1, POU2F3 and YAP1 could provide a reproducible method of classifying SCLC for this scope, considering that cMyc tends to be overexpressed in SCLC-N subtype.

## Author contributions

ASte and GP did write the review, FS, AStr, EV, SF, DB, FM, IS GV, and MF did review all the specific literature and pooled all available data from peer-reviewed journal and featured oncology meetings, ED’A, MDS, and CC did critically review all the drafts and GT and EB did coordinate the whole work. All authors contributed to the article and approved the submitted version.

## Funding

EB is supported by Institutional funds of Università Cattolica del Sacro Cuore (UCSC-projects D1) and by the Fondazione AIRC (Associazione Italiana Ricerca sul Cancro) under Investigator Grant (IG) No. IG20583.

## Conflict of interest

EB received advisory and speakers’ fee from MSD, Astra-Zeneca, Celgene, Pfizer, Helsinn, Eli-Lilly, BMS, Novartis, and Roche.

The remaining authors declare that the research was conducted in the absence of any commercial or financial relationships that could be construed as a potential conflict of interest.

## Publisher’s note

All claims expressed in this article are solely those of the authors and do not necessarily represent those of their affiliated organizations, or those of the publisher, the editors and the reviewers. Any product that may be evaluated in this article, or claim that may be made by its manufacturer, is not guaranteed or endorsed by the publisher.
